# Differential parasitism by four species of phorid flies when attacking three worker castes of the leaf-cutting ant *Atta laevigata* (Smith, 1858)

**DOI:** 10.1371/journal.pone.0250973

**Published:** 2021-05-05

**Authors:** Maria Lucimar O. Souza, Rafael J. Oliveira, Danival J. Souza, Richard I. Samuels, Marcos A. L. Bragança

**Affiliations:** 1 Instituto Federal de Educação, Ciências e Tecnologia do Tocantins, Curso de Gestão em Agronegócio, Palmas, Tocantins, Brazil; 2 Universidade Federal do Tocantins, Curso de Ciências Biológicas, Porto Nacional, Tocantins, Brazil; 3 Universidade Federal do Tocantins, Curso de Engenharia Florestal, Gurupi, Tocantins, Brazil; 4 Universidade Estadual do Norte Fluminense Darcy Ribeiro, Rio de Janeiro, Brazil; Universidade Federal de Ouro Preto, BRAZIL

## Abstract

Certain species of parasitic flies belonging to the Phoridae are known to attack *Atta* spp. workers foraging along trails, near nest openings used by the ants to supply the colony with plant material, and in the areas where the ants are actively cutting plant material. However, there have been no previous studies of phorid parasitism of non-foraging worker ants, for example excavators and soldiers. Excavators can be found on the surface around specialized nest openings, carrying and dumping soil on characteristic mounds. Soldiers can be found on the trails protecting foragers or guarding the different types of nest openings. The current study was performed to investigate the differential parasitism rates of *Atta laevigata* (Smith, 1858) worker castes by four species of phorids. Ants of all castes on trails and at nest entrances were collect from 18 mature colonies in the field. A total of 21,254 ants were collected from trails and 14,649 collected from the mounds of loose soil near nest openings. The captured workers were maintained under controlled laboratory conditions to evaluate the rate of parasitism. Of the ants collected from trails, 1,112 (5.23%) were found to have been parasitized, of which 1,102 were foragers and only 10 were soldiers. Of the ants collected from the soil mounds near the nest openings, only 27 (0.18%) were found to have been parasitized, of those 25 were excavators and 2 were soldiers. When evaluating parasitism of ants on the trails, 46.2% were attacked by *Apocephalus attophilus* Borgmeier, 1928, 22.6% by *Myrmosicarius grandicornis* Borgmeier, 1928, 16.6% by *Eibesfeldtphora erthali* (Brown, 2001) and 14.6% by *Apocephalus vicosae* Disney, 2000. Only two species of phorid, *M*. *grandicornis* and *E*. *erthali*, were observed parasitizing excavators, whilst only *E*. *erthali* parasitized soldiers. This is the first time that *Atta* spp. excavators and soldiers have been shown to be parasitized by phorids. The low rates of parasitism and specificity of certain phorid species for excavators and soldiers is discussed in relation to the behavioral interactions of hosts and their parasitoids, as well as the relationship between host and parasitoid size.

## Introduction

Leaf-cutting ants of the genera *Atta* Fabricius, 1804 and *Acromyrmex* Mayr, 1865 are the dominant herbivores in many Neotropical habitats, forming a monophyletic group [1–3]. They specialize in cutting and collecting fresh plant material that is used to cultivate a symbiotic fungus inside their nests. The symbiotic fungus provides the sole nutrient source for ant larvae and queens [4,5], as well as supplementing the diet of worker ants [6]. The habit of building large underground nests results in the excavation of large volumes of soil and the movement of large quantities of plant material from the surface to the underground fungus garden chambers. These ants play several important roles in natural ecosystems, such nutrient recycling, seed dispersal, as well as improving the physical and chemical properties of the soil [7–9].

*Atta laevigata* (Smith, 1858) and *Atta sexdens* (Linnaeus, 1758) have a wide ranging distribution in Brazil and are the main pests of a large number of economically important plant species. They cause damage in agricultural, forestry and urban areas, resulting in significant losses due to their leaf-cutting activities [3,10]. The most commonly used method to control these two species is the application of insecticidal granulated baits. These insecticides are, in general, highly polluting for the soil and unspecific, that is, they have the potential to kill several non-target species [11–13]. In the search for alternative methods of control, it is of great interest to investigate the potential of natural enemies to regulate leaf-cutting ant populations [3,14]. In that respect, small parasitic flies of the family Phoridae may have potential for use in biological control programs against leaf-cutting ants.

The Phoridae consists of thousands of different species, with a wide range of larval habits. The larval stages of some species are saprophytic or herbivorous, but the majority are predatory or specialized parasitoids [15]. In many cases, phorids parasitize social insects, especially ants [15,16]. The interaction between *Pseudacteon* spp. Coquillett, 1907 and fire ants of the genus *Solenopsis* Westwood, 1840 has been extensively studied in recent decades [17–20], and currently these flies are produced in the laboratory and released in the field for the classical biological control of *Solenopsis invicta* Buren, 1972 [21]. In recent decades, studies of the biological and behavioral characteristics of parasitic phorids of the genera *Eibesfeldtphora* Disney, 1996, *Myrmosicarius* Borgmeier, 1928 and *Apocephalus* Coquillett, 1901, which attack leaf-cutting ants of the genera *Atta* and *Acromyrmex*, have intensified [22–26]. The presence of phorid flies on the trails has also been observed to decrease the foraging rates of two species of leaf-cutting ants, *A*. *sexdens* and *Atta vollenweideri* Forel, 1893 [27–29].

Studies have shown that female phorids parasitize foraging *Atta* spp. workers [23,27,30,31]. The phorids attack these ants on the trails, whilst they are cutting plant material or transporting the leaves back to the nest. Foragers are responsible for locating, cutting and transporting plant material to the interior of the nests [1,32]. The phorids land on a preferred host ant and lay their eggs in specific regions of the head or abdomen [33–35]. During the attacks, which usually only last for about one or two seconds, the females of most phorid species lay a single egg inside the host’s body (endoparasitoidism), with a single larva developing inside the host, normally resulting in host death [36–39]. Studies have shown that phorid parasitism of foraging *Atta* spp. workers on trails ranged from less than 1% to over 30% [23,33,35,40,41], with parasitism of *A*. *laevigata* ranged from 2.8% to 5.3% [33,35,42].

In addition to trails and foraging areas, phorid attacks can occur close to the nest openings through which foragers enter the nest carrying fresh plant material [28]. These openings can be distributed at different distances around the nest. *Atta laevigata* foraging trails are, in general, physically well defined, leading from the principal nest supply orifices to the areas where the ants are cutting leaves [43,44].

Typically, the externally visible part of a *A*. *laevigata* nest consists of a large mound of loose soil with several entrance holes leading to subterranean underground chambers connected by tunnels. The symbiotic fungus is cultivated in some of these underground chambers, whilst other chambers are used to deposit unwanted residues. The characteristic mound of loose soil around *Atta* spp. nests is formed by the soil excavated following the construction of tunnels and chambers, which is transported to the surface [44]. The soil is excavated by the so called “excavator” worker caste, which Wilson [1] defined as “forager-excavators” in *A*. *sexdens* colonies. The excavated material consists of agglomerates of a mixture of soil, humus and litter, which is carried between the jaws of the ants in the form of pellets and deposited externally near the exit holes, forming a pile of loose material. Each *A*. *laevigata* nest orifice used by the excavators is normally located in the center of a crater or at the bottom of a small flattened funnel or so called “volcano” [32,43]. Although a study of *A*. *vollenweideri* divided tunnel workers into two types: “excavators” which are directly involved in excavation activities and “loaders” that transport the pellets to the surface [45], in the current study, the *A*. *laevigata* workers that carried the material to the surface and observed on the soil mounds were denominated as “excavators”.

Wilson [1] defined four castes of *A*. *sexdens* workers according to their role in the colony: gardener-nurses, within-nest generalists, forager-excavators, and defenders. Although in *A*. *sexdens*, foraging workers and the excavator-workers were both included in a single size group by Wilson [1], with a mean cephalic capsule width of 2.2 mm, in the present study preliminary observations indicated that in *A*. *laevigata*, the foragers have larger head capsule widths than excavators. Therefore, differences between castes could be based not only on the different tasks performed in the colony but also on worker size.

On occasions, the orifices which lead from the nest chambers to the soil mound can be used by ants to carry plant material into the nests and foraging *A*. *laevigata* workers that transit through these opening at this time can also be attacked by phorids [22,34,42]. However, there are no reports in the literature of phorids attacking the excavator caste of any *Atta* species. Likewise, phorid parasitism of the worker caste denominated as “soldiers” has not been documented [23,24]. *Atta* soldiers commonly transit through the openings on the loose soil mound, through the main nest supply openings and along trails, protecting the ants from enemies. The soldiers are the largest workers in *Atta* spp. colonies [46]. Furthermore, as *Atta* workers are highly polymorphic [47], certain species of phorids have evolved host size preferences and it is possible to observe distinct host species-parasitoid species interactions [26,28,34,41,48,49]. Therefore, worker castes of different sizes, for example, foragers, excavators and soldiers, could be parasitized by different sets of phorid species with different parasitism rates.

Larger (non-parasitized) *Atta* workers typically survive for longer in the laboratory than smaller workers (MALB, personal observation). However, due to the detrimental effects on host physiology, parasitized individuals of all size groups survive for less time than their non-parasitized counterparts and the survival time of parasitized worker ants can depend on the species of phorid with which they have been parasitized [22,35]. Therefore, it is logical to assume that the life span of an individual host ant will determine whether or not the parasitoid will have sufficient time to develop inside that host.

In the present study we investigated whether *A*. *laevigata* excavator and soldier ants were parasitized by phorid flies and if there was a distinct set of species attacking these castes when compared to foragers. We also investigated the survival of different worker castes when parasitized by different phorid flies. Further studies were also carried out to investigate the interactions in relation to host size and parasitoid size. This information may be useful for the planning of biological control strategies and aid the further understanding of the biodiversity and biology of phorids associated with leaf-cutting ants.

## Materials and methods

**Field Work Permit**: Ministry of the Environment—IBAMA number: 12433–3.

### Study area

This study was carried out in the municipality of Palmas (10°14’56’’S; 48°19’29’’W), in the state of Tocantins, Brazil, between 2018 and 2019. Palmas is located in a tropical climate region with a well-defined dry season in the winter. The rainy season extends from October to April, whilst the dry season occurs from May to September. The temperature of the region has an isothermal characteristic, with differences in the average temperatures between the warmest month and the coldest month being less than 5°C [50]. The predominant vegetation cover in Palmas is “Cerrado *stricto sensu*”, being a region characteristic of open savannas with patches of gallery forests.

Eighteen *A*. *laevigata* nests located in public and private sites in an urban region of Palmas were studied. Seven nests were located at the Palacinho sample site (PAL), six at the EMBRAPA site (EMB) and five at the University of Tocantins (UFT) site. The distance between PAL and EMB sites was approximately 4 km, whilst the distance between the UFT and EMB sites and between the UFT and PAL sites was approximately 6 km. The EMB site was located in an area composed of a vegetation structure characteristic of Cerrado *stricto sensu*, with small trees, with twisted stems. The grass stratum is discontinuous and small (30 cm), with several regenerating species, herbs and small shrubs that intertwine with the trees and shrubs of the woody stratum. The PAL site was located in an area occupied mainly by tree species exotic to the Cerrado, which are widely used in the urban forestation in Palmas. However, species native to the region’s Cerrado, including some fruit trees, were also present. The UFT site had a predominance of species that are exotic to the Cerrado, originating from other regions in Brazil or other countries.

The vegetation of this biome undergoes direct changes as a result of the climatic variations that occur during the different months of the year, presenting desert characteristics during the dry period and rapid vegetative growth during the rainy period [51].

### Collection of *Atta laevigata* workers

**Fig 1:**
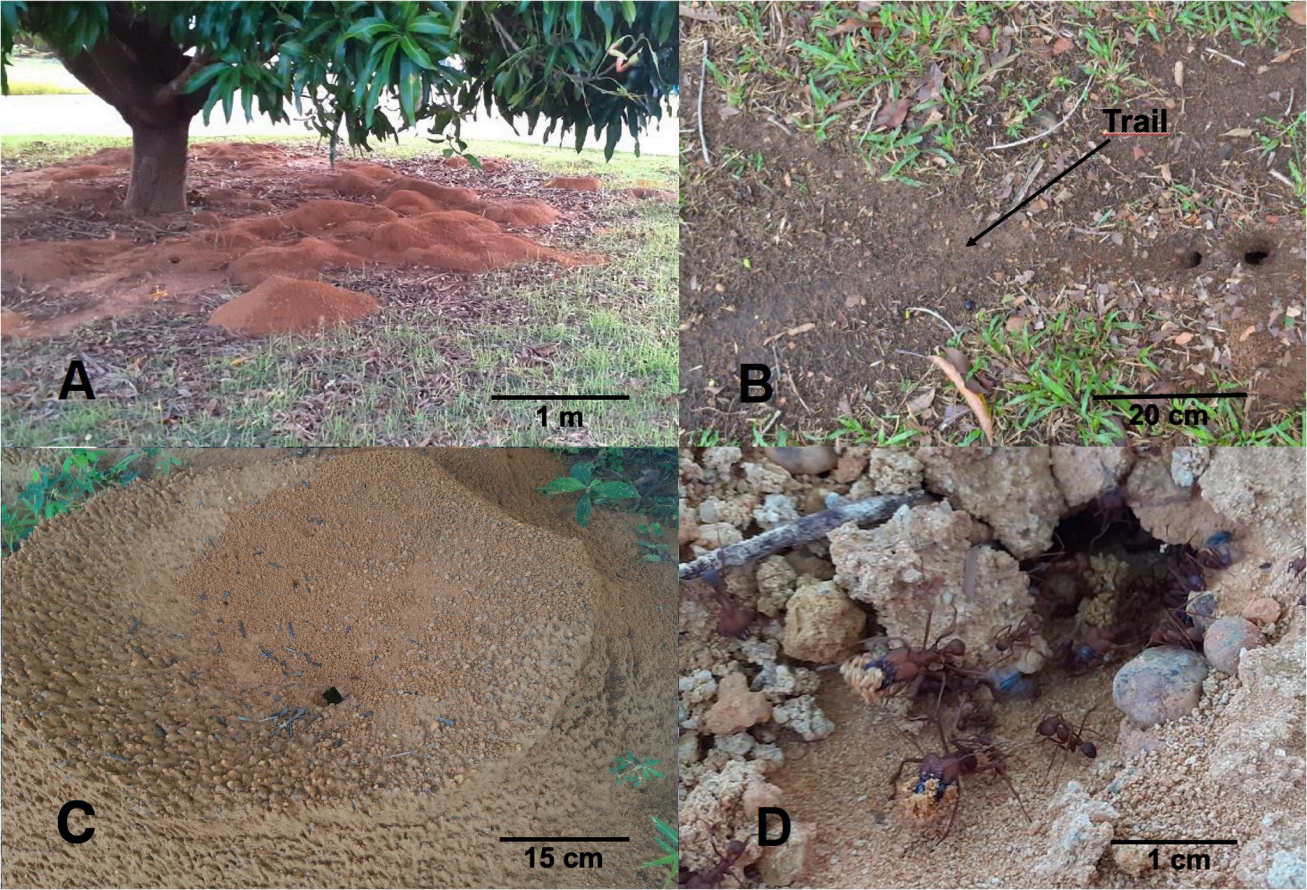
**A**: *Atta laevigata* nest; **B**: Supply orifice with foraging trail; **C**: A crater with an orifice in the center; **D**: Details of excavator workers carrying pellets (mixture of soil, humus and litter) with their mandibles. Photos: M.L.O. Souza.

Six collections of *A*. *laevigata* workers were carried out from the seven nests located at the PAL site at intervals of approximately 15 days between February and May 2018. Between June and September 2018, five collections were carried out every 15 days from the six nests selected at the EMB site. Then, between October 2018 and January 2019, four more collections, at 25-day intervals, were carried out from the five nests selected at the UFT site. The collections were nocturnal and started between 18:00h and 19:00h, with the ant collection sessions from each colony lasting approximately 20–25 min. During this period, foraging worker ants found on one or two trails of 2 to 5 m in length originating from each nest (Fig 1A and 1B) were randomly captured with the aid of flexible metal tweezers and placed in a plastic pot (9 x 14 cm). Likewise, soldiers on the trails were captured and placed in a separate pot. During the same 20–25 min period, excavator workers and soldier ants passing through the openings in two craters on the loose soil mounds (Fig 1C and 1D) were captured and placed separately in two other pots. Not all of the colonies were sampled during the collections at the same site, as sometimes there was no flow of ants on the trails or over the soil mounds. Inert non-toxic talc was placed around the edges of each pot to prevent ants from escaping during collection. Lids were then placed on the pots, which were taken to the Entomology Laboratory, Biological Collections Building at the Federal University of Tocantins, Palmas University Campus, Tocantins, Brazil.

### Ant maintenance and obtaining parasitoids

Ants were placed in four plastic trays (55 x 34 x 9.5 cm) to which talc had been applied around the inner edges of the trays to prevent escape and covered with perforated lids to allow air circulation. The trays were placed in an incubator (26 ± 1°C, 85 ± 5% RH) and the ants were offered a 50% honey solution soaked in cotton swabs, which were changed every two days [22,35]. During the next 15 days, each tray was inspected daily and dead ants were removed. Dead ants were transferred to individual glass tubes (12 x 75 mm) plugged with cottonwool and maintained in an incubator as stated above. A 15 day period was chosen as this was the maximum time period for phorids to kill their hosts [28,33,37]. On the 15th day, any individuals (non-parasitized) that were still alive were placed in 70% alcohol. The number of surviving ants (non-parasitized) and those which had died up to day fifteen (parasitized and non-parasitized individuals) was used to determine the total number of workers collected from the mounds and trails for each collection.

The number of workers from trails per collection at the PAL site ranged from 102 to 739, whilst between 81 to 531 ants were collected from the mounds at the same site. The number of workers per collection on the trails at the EMB site ranged from 37 to 718, whilst 37 to 290 ants were collected from mounds. At the UFT site, these numbers ranged from 335 to 739 on the trails and from 216 to 820 on the mounds. The fluctuation in the numbers of workers captured on trails and mounds during the 15 collections was due to the natural variation in the activity of workers when comparing colonies and collection days.

Dead individuals were maintained in an incubator for 24 h in order to differentiate between parasitized and non-parasitized ants. It was possible to observe phorid larvae or pupae developing inside the hosts bodies [28,35,52]. Parasitized ants were kept in the tubes in the incubator until the flies emerged from the hosts. After emergence, the flies were maintained in the chamber for another 24 hours to allow for sclerotization of the recently formed phorid integument.

The flies were then individualized in Eppendorf tubes containing 70% alcohol and labeled with the host collection site (soil mound or trail) and worker caste host (excavator, forager or soldier). The parasitized worker ants from which flies had emerged and those from which no parasitoids had successfully emerged, were stored individually in paper envelopes. A similar procedure was also performed for workers that died without any signs of parasitism, except that they were stored in envelopes in cohorts relative to the day of death. The taxonomic identification of the phorids was performed according to published keys [53–57]. The flies and their host ants were deposited in the UFT Entomology Laboratory insect collection.

### Measurements of ants and phorids

The size of the ants collected from random samples of parasitized and non-parasitized workers (excavators, foragers, soldiers on mounds or on trails) was measured. The head capsule width (mm) at the widest point was determined [1]. Adult phorid size was estimated by measuring the length (mm) of one of the wings from each individual [58]. All measurements were performed using a stereomicroscope with a calibrated (millimeter) graticulated eyepiece.

### Ant survival time

The average survival time of parasitized worker ants (foragers, excavators and soldiers) was calculated from the day of collection until the day of death. Based on the literature, no parasitized ants survived for more than 15 days after collection. End point mean survival times of non-parasitized workers was calculated over the first 15 days following collection as the aim of this experiment was not to evaluate total survival times of these ants in the laboratory. For parasitized insects, the phorid species which emerged from each host was recorded to correlate this data with ant survival time.

### Data analysis

The parasitism frequency of worker of ants collected on trails or from soil mounds were calculated as a percentage of the total number ants collected in relation to the number which were subsequently observed to have been parasitized. The proportional parasitism rate (%) was calculated considering the percentage with which each species of parasitoid contributed to the parasitism of *A*. *laevigata* workers on the trails and mounds. This rate was also calculated when considering the species of parasitoid that emerged from workers of different castes.

Despite transforming the original data using arcsin √(%/100), the mean parasitism rates of foragers did not follow a normal distribution and therefore the Kruskal-Wallis (H) test was applied, followed by Dunn’s multiple comparison test to assess the statistical differences in the parasitism rates between different phorid species. Although there was a large variation in the number of workers collected from trails and mounds when considering collection days and different sites, statistical analysis comparing parasitism rates between worker castes and between parasitoid species was possible because percentage parasitism was used and not numerical values.

The average survival times of non-parasitized foragers, excavators and soldiers (from mounds and trials) and the effect of parasitism on survival time of workers when parasitized by different phorid species was analyzed using the Kruskal-Wallis (H) test, followed by the Dunn’s multiple comparison test. The H test was used to compare survival times because this data did not fit a normal distribution despite transformation of the data.

ANOVA and the Tukey tests for the separation of means were performed to compare the head capsule width (mm) of the following groups of workers: (i) excavators, foragers, soldiers on trails and soldiers on mounds without parasitism; (ii) comparing the set of excavators and foragers with and without parasitism; (iii) parasitized foragers and excavators; (iv) to compare ants parasitized by different species of phorid flies. ANOVA and Tukey’s tests were also applied to compare phorid adult wing length (mm). Assuming homogeneity of variances and to adjust the data to a normal distribution for the validation of ANOVA, the original data for the measurements of the ant head capsule width and phorid fly wing length were transformed using box-cox. This procedure can be used to estimate the best transformation to achieve normality [59].

The means are shown with their respective standard deviations or confidence intervals. All analyzes were conducted using the R package [60], with the null hypotheses tested at the 5% probability level.

## Results

A total of 35,903 *A*. *laevigata* worker ants were collected during the study period from the three sample sites (PAL, EMB and UFT). Worker ants from all three sites were found to have been parasitized by four species of Phoridae. One thousand one hundred and forty-one ants (3.17% of the total number collected) were observed to have been parasitized. Of those, 1.43% were parasitized by *Apocephalus attophilus* Borgmeier, 1928 (n = 514), 0.74% by *Myrmosicarius grandicornis* Borgmeier, 1928 (n = 266), 0.55% by *Eibesfeldtphora erthali* Brown, 2001 (n = 197) and 0.45% by *Apocephalus vicosae* Disney, 2000 (n = 162) (Table 1). Only one phorid fly emerged from each of the worker ants parasitized by *M*. *grandicornis*, *A*. *vicosae* and *E*. *erthali*. However, for ants parasitized by *A*. *attophilus*, one to fourteen flies emerged from individual hosts, with the mean number of parasitoids per host being 3.1 (± 2.74 SD).

**Table 1:** Total and proportional parasitism rates of four species of phorid flies attacking *Atta laevigata* workers (foragers, excavators and soldiers) collected on trails or from soil mounds.

LOCAL Worker caste	Number of workers collected	Number of parasitized workers	Parasitism rate (%)	Number of parasitized workers (n) and proportional rate of parasitism (%)							
				*Apocephalus attophilus*		*Myrmosicarius grandicornis*		*Eibesfeldtphora erthali*		*Apocephalus vicosae*	
				n	%	n	%	n	%	n	%
**TRAIL: TOTAL**	21,254	1,112	5.23	514	**46.22**	251	**22.58**	185	**16.63**	162	**14.57**
Foragers	15,757	1,102	7.00	514	*100*	251	*100*	175	*94*.*60*	162	*100*
Soldiers	5,497	10	0,18	0	*0*	0	*0*	10	*5*.*40*	0	*0*
**MOUND: TOTAL**	14,649	27	0.18	0	0	15	**55.55**	12	**44.45**	0	0
Excavators	10,631	25	0.23	0	0	15	*100*	10	*83*.*33*	0	0
Soldiers	4,018	2	0.05	0	0	0	*0*	2	*16*.*67*	0	0

A total of eighteen colonies were sampled. The proportional rates of parasitism were calculated as a ratio of the number of parasitized ants (n) when comparing different species of phorids (% bold type) on trails and mounds, or for each phorid species when comparing different worker castes on trails or on mounds (% italics).

The total number of workers which had been parasitized on the trails (foragers + soldiers) was 1,112, with only 27 excavators and soldiers collected from the mounds found to have been parasitized. The separation of the results into workers on trails versus workers on mounds showed that the parasitism rate, when only considering the total number of ants on the trails, increased to 5.23%, which was much higher that seen for the ants collected only from the mounds (0.18%) (Table 1). Ten of the soldiers captured on the trails and two on the mounds had been parasitized by *E*. *erthali*, which was the only parasitoid associated with this caste. Foraging workers were parasitized by all four species of phorids identified in this study, with the highest proportional rate of parasitism seen for *A*. *attophilus* (46.22%), followed by *M*. *grandicornis* (22.58%). For *E*. *erthali* and *A*. *vicosae*, lower rates of 16.63% and 14.57% were observed, respectively (Table 1). When considering parasitism of the excavator caste, only fifteen individuals had been parasitized by *M*. *grandicornis* and ten by *E*. *erthali*. There was a significant difference in the average rates of parasitism of foragers by the four species of parasitoids (H = 44.29; n = 272; p <0.001). These rates were similar and not significantly different using Dunn’s multiple separation test (p >0.05) when comparing *A*. *attophilus* and *E*. *erthali*, but the rates for both of these species were significantly higher (p <0.001) than those for *A*. *vicosae* and *M*. *grandicornis*. There was no significant difference between the parasitism rates of the later two species of phorids.

Parasitized ants survived for a maximum of 10 days (Table 2). Non-parasitized worker ant survival was only evaluated over a 15 day period in the laboratory after collection in the field. There was no significant difference in the mean survival of non-parasitized excavators and foragers (5 days), however, on average these ants survived three days less than soldiers collected on mounds or on the trails, which had an average life-span of 8 days following collection (Table 2). There was no significant difference in the survival times of non-parasitized soldiers collected on trails or mounds. When considering the survival of parasitized workers (of all castes and from all locations), those that had been parasitized by *A*. *attophilus* survived for significantly less time (2.8 days) than the workers parasitized by the other three phorids, while the workers parasitized by *M*. *grandicornis* survived the longest after collection (6 days) (Table 2).

**Table 2:** Mean survival times of parasitized and non-parasitized *Atta laevigata* workers, survival times of different non-parasitized castes and worker survival time in relation to phorid species.

Workers	Survival (days)				
	Mean ± SD	Number of workers	Range (minimum to maximum)	Kruskal-Wallis test (H)	p-value
Parasitized	4.0 ± 2.1ª	1137	1 to 10	497.50	< 0.001
Non-parasitized	6.7 ± 3.3^b^	1783	1 to 14		
**Non-parasitized**:					
Excavators	5.6 ± 3.1^a^	487	1 to 14	287.69	< 0.001
Foragers	5.4 ± 3.3ª	454	1 to 14		
Soldiers (mound)	8.1 ± 2.7^b^	389	1 to 14		
Soldiers (trail)	8.2 ± 2.9^b^	453	1 to 14		
**All castes parasitized by**:					
*Apocephalus attophilus*	2.8 ± 1.1ª	512	1 to 7	411.35	< 0.001
*Apocephalus vicosae*	4.2 ± 1.5^b^	162	1 to 9		
*Eibesfeldtphora erthali*	4.1 ± 2.4^c^	197	1 to 9		
*Myrmosicarius grandicornis*	6.1 ± 1.9^d^	266	2 to 10		

Note: Different letters indicate significant differences using Dunn’s multiple comparison test at the 5% level.

**Fig 2:**
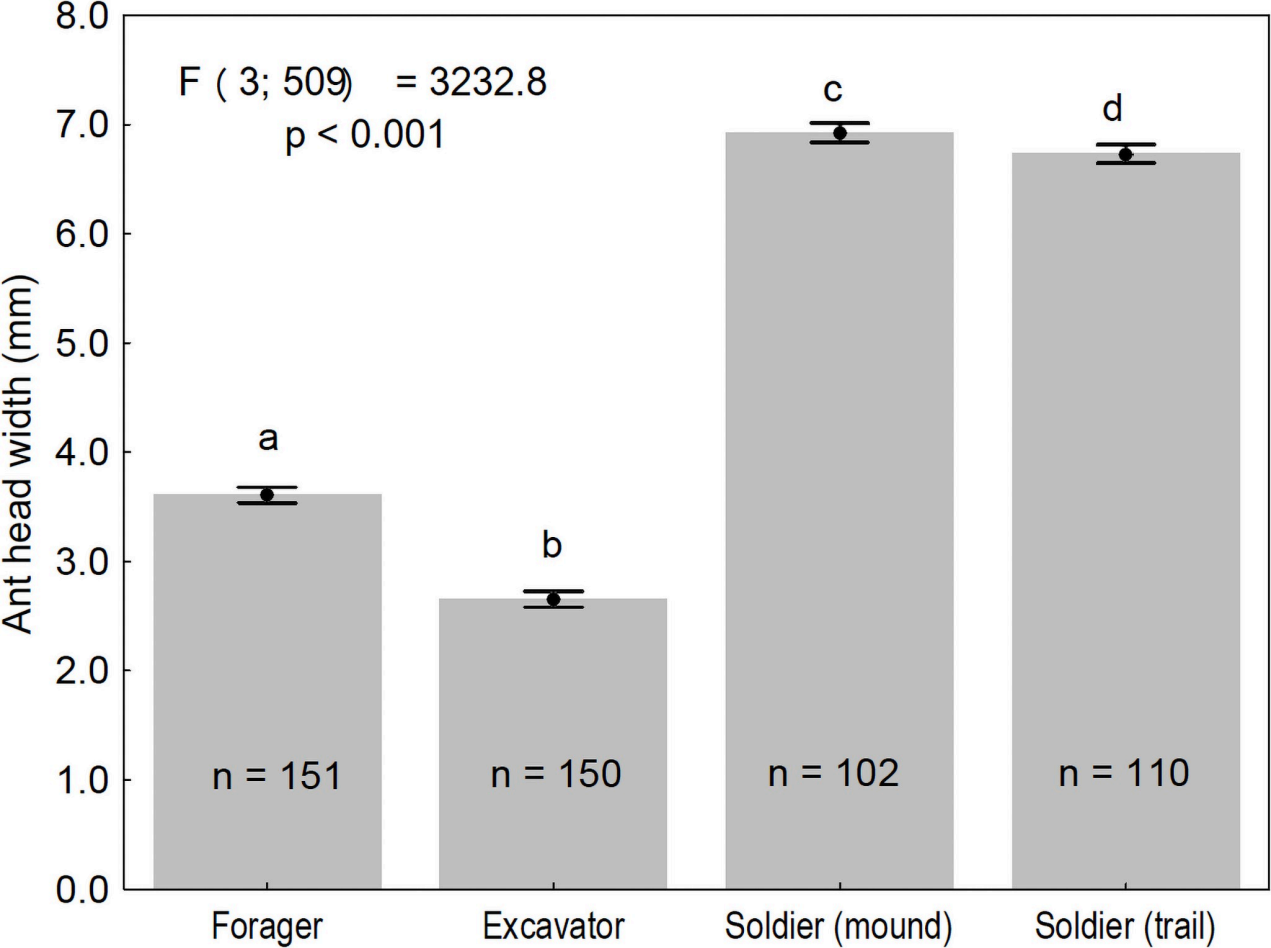
Comparison of head capsule width of non-parasitized *Atta laevigata* workers collected from mounds (excavators and soldiers) or from trails (foragers and soldiers). Note: Vertical bars indicate 95% confidence intervals, and different letters indicate significant differences between mean head capsule widths (one-way ANOVA and Tukey’s test at 5% level). The original data was transformed using box-cox (λ = 1.35).

The data showed that the largest workers collected from *A*. *laevigata* colonies were of the soldier caste. Soldiers on mounds were significantly larger than on trails (Fig 2). On average, soldiers were more than twice the size of the foragers and excavators (Fig 2). Foragers collected from trails were significantly larger than the excavators (Fig 2). The average size of non-parasitized foragers and excavators when considered together (3.13 ± 0.69 mm; n = 301) was greater (F_1;952_ = 17.48; p <0.001) than parasitized workers (2.92 ± 0.73 mm; n = 653). When considering parasitized ants, the size of foragers (2.94 ± 0.73 mm; n = 628) was significantly larger (F_1;651_ = 11.35; p = 0.001) than excavators (2.44 ± 0.76 mm; n = 25).

**Fig 3:**
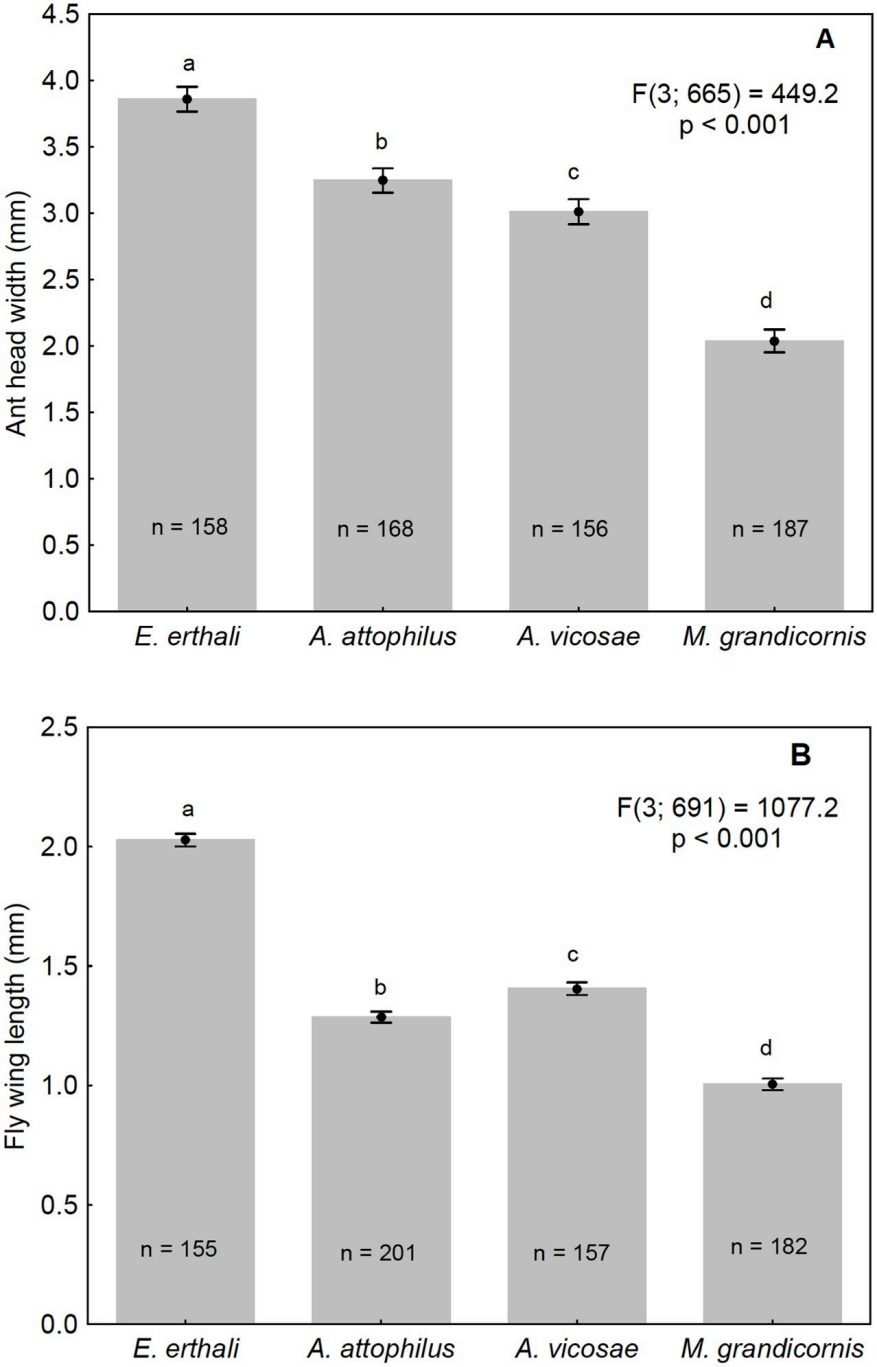
(A) Relationship between *Atta laevigata* worker size and the phorid species parasitizing these ants; (B) Size (wing length) of the four species of phorids. Note: Vertical bars indicate 95% confidence intervals and different letters indicate significant differences in ant cephalic capsule width and phorid wing length. A one-way ANOVA and the Tukey test at the 5% level were used for comparisons. Data was transformed using box-cox: λ = -0.30 (A); λ = -0.55 (B).

**Fig 4:**
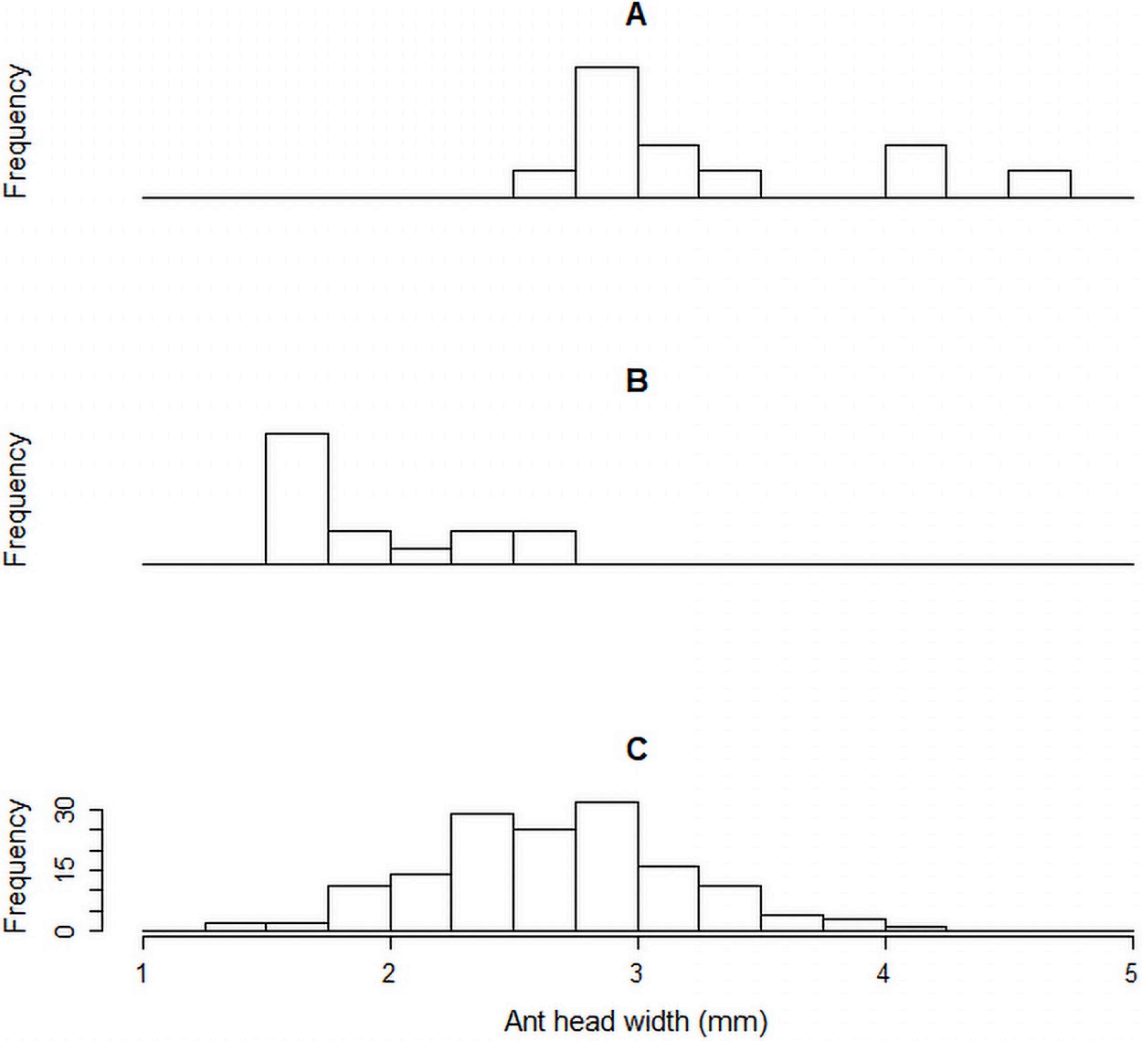
*Atta laevigata* excavator workers size (head capsule width) in relation to frequency of the two species of phorids parasitizing this caste. **A**: *Eibesfeldtphora erthali* (n = 10); **B**: *Myrmosicarius grandicornis* (n = 15); **C**: Size range of excavators (n = 150) collected on the mounds of loose soil near the nests.

When considering parasitized *A*. *laevigata* worker ants in general, those with the largest average head capsule widths were observed to have been preferentially parasitized by *E*. *erthali*. Slightly smaller workers were parasitized by *A*. *attophilus* and the smallest ants were parasitized by *M*. *grandicornis* (Fig 3A). The phorids that emerged from the largest and smallest hosts, that is, *E*. *erthali* and *M*. *grandicornis* respectively, were also the largest and smallest flies when considering average wing length as a parameter (Fig 3B). However, there was an inversion in the order of parasitoid size (wing length) when considering *A*. *vicosae* and *A*. *attophilus* in relation to the size of their hosts (Fig 3A and 3B). Although *A*. *attophilus* flies are smaller than those of *A*. *vicosae*, they emerged from larger hosts than those of *A*. *vicosae*. The results in Fig 4 demonstrated the relationship between the size of the excavators and the frequency that the two specific parasitoid species attacked excavators. *Eibesfeldtphora erthali* was observed to prefer larger excavators (Fig 4A), whilst *M*. *grandicornis* parasitized smaller ants of this caste (Fig 4B).

## Discussion

Four species of parasitic phorids were observed parasitizing *A*. *laevigata* in this study, in which the rate of total parasitism of this host was similar to that of other studies of *Atta*. Foraging workers and soldiers on trails had parasitism rates that were much higher than excavator workers and soldiers from mounds. Foragers were hosts to all four species of parasitoid, while excavators were parasitized by two species (*E*. *erthali* and *M*. *grandicornis*) and the soldiers were only parasitized by *E*. *erthali*. The hypothesis that parasitism of excavators was low or absent when compared to foragers workers because the excavator’s life span is too short for the development of the parasitoids within this host was rejected. However, the low rates of parasitism of excavators and soldiers by *E*. *erthali* and *M*. *grandicornis*, and the fact that these castes were not parasitized by *A*. *attopphilus* and *A*. *vicosae* were justified based on the results for the relationship between parasitoid and host size and on other studies which observed the attack behavior of phorid flies in relation to the different tasks performed by the ants.

Among the three genera of Phoridae studied here, *Eibesfeldtphora* is considered to be a specialist parasitoid of the genus *Atta*, with fewer species found parasitizing *Acromyrmex* [22,24,61]. On the other hand, species of *Myrmosicarius* can also parasitize other ant genera, such as *Solenopsis* and *Labidus* Jurine, 1807 [55]. *Apocephalus* is the most diverse of the three genera, mainly parasitizing *Acromyrmex* spp., but also *Atta*, *Pheidole* Westwood, 1839, *Camponotus* Mayr, 1861, *Pachycondyla* Smith, F. 1858, *Ectatomma* Smith, F. 1858, *Paraponera* Smith, F. 1858 among others [62–64].

The four species of phorids that parasitized *A*. *laevigata* described in the current study were also found in another region of the Cerrado biome parasitizing *A*. *laevigata* and *A*. *sexdens* [22]. In the Atlantic Forest biome in Brazil, *A*. *laevigata* and *A*. *sexdens* are also hosts to all four parasitoids described in the present study [28,33,61,65], whilst *Atta bisphaerica* Forel, 1908 is parasitized by *M*. *grandicornis* and *A*. *attophilus* [55,66,67]. The total parasitism rate of *A*. *laevigata* by phorids seen here (3.17%) was higher than that recorded for the same host (2.8%) by Bragança & Medeiros [35], but lower than the rates (4.0%) recorded in the studies by Erthal & Tonhasca [33] or by Bragança et al. [22] (5.36%). The current results for *A*. *laevigata* parasitism rates by phorids are within the range commonly found for *Atta* spp., of which the lowest documented was <1% and the highest approximately 6%. In *A*. *sexdens*, for example, four different studies reported rates of 0.8% [42], 1.57% [22], 2.94% [49] and 2.6% [68]. The parasitism rates by phorids recorded in two studies of *A*. *bisphaerica* were 1% and 4.4%, respectively [66,69].

In the aforementioned studies, the evaluation of *Atta* spp. parasitism rates by phorids was carried out by sampling only foraging workers transiting along the trails or foragers that were collected from the nest supply orifices. No previous studies have evaluated the parasitism of *Atta* spp. excavators observed on the mounds of loose soil around the specialized nest orifices through which these excavator ants carry material to the surface. In the current study 18 colonies were sampled in order to collect not only the foragers on the trails but also the excavators (from mounds) and soldier ants (from mounds and trails). Interestingly, the parasitism rate of the excavators was 30 times lower than that seen for the foragers. Phorid parasitism of *A*. *laevigata* soldiers was also documented here for the first time, although similarly to the excavators, the parasitism rates were very low when compared to foragers. In fact, the number of soldiers that were parasitized was half that of the excavators. Furthermore, the number of soldier ants collected from mounds which had been parasitized was even lower than the number of parasitized soldier ants collected on trails. The information available from previous studies on *A*. *vollenweideri* [23,24], *A*. *laevigata* and *A*. *sexdens* (MALB, personal observation) indicated that phorid flies do not parasitize the soldier caste.

The distribution of phorid parasitism, when considering the four parasitoid species studied here, showed that attacks by *A*. *attophilus* were the most frequent (46%). This result was similar to that seen in previous studies of *A*. *laevigata* and also for other species of leaf-cutting ants such as *A*. *sexdens* and *A*. *bisphaerica* [22,66,68,69]. In fact, the ability of *A*. *attophilus* to cause high levels of mortality differentiated it from other species of parasitoids. Biological characteristics such as a highly successful emergence rate (93%) from hosts and the production, on average, of 2.5 individuals per host ant has been observed when *A*. *attophilus* attacked *A*. *bisphaerica* [66]. *Eibesfeldtphora bragancai* (Brown, 2001 [73]) and *M*. *grandicornis* parasitizing *A*. *bisphaerica* showed emergence rates of 41 and 57%, respectively, with production of a single parasitoid per host [66].

In the current study, an average of three *A*. *attophilus* (ranging from one to fourteen parasitoids) emerged from each host forager ant, whilst the other three species of phorids had a solitary habit, laying only one egg in each host insect. These characteristics highlight the potential of *A*. *attophilus* as a biological control agent against leaf-cutting ants [66,68]. Also in the current study, foragers that had been parasitized by *A*. *attophilus* had the shortest survival time (2.8 days) following collection in the field. This was probably due to the rapid development of this parasitoid within the host forager. On the other hand, *A*. *attophilus* did not parasitize excavators. Here we observed that only *M*. *grandicornis* and *E*. *erthali* parasitized the excavator caste.

The reduced lifespan of parasitized ants was not unexpected as the development of parasitoid larvae would have a detrimental effect on the hosts. A similar result has been observed for phorids attacking *A*. *laevigata* [35]. However, the similar survival times of non-parasitized ants from the trails (foragers and soldiers) and earth mounds (excavators and soldiers) is not related to the high parasitism rates of foragers and trail soldiers compared to excavators and soldiers from mounds.

In the leaf-cutting ants it has been observed that the younger workers tend to perform tasks in the fungus gardens, while the older workers perform tasks outside the nest, precisely those that represent higher risks of mortality [70,71]. It is thought that excavators maybe older than foragers and, therefore, their remaining life span, from time of parasitoid oviposition, could be less than the time necessary for the parasitoid to complete its development in the host’s body. Thus, even if the attack and oviposition behaviors against excavators were the same as those performed against foragers, excavator parasitism rates would therefore be lower or the parasitism would not be successful as the hosts would die before the parasitoids completed larval and pupal development.

In addition, even considering that the workers’ life span was recorded for only 14 days, as on the 15^th^ day those that remained alive were sacrificed, the average survival of the non-parasitized excavator workers (mean 5.6 days; range: 1–14) and the mound soldiers (mean 8.1 days; range: 1–14) when compared to the survival of ants parasitized by *A*. *attophilus* (mean 2.8 days; range: 1–7) and *A*. *vicosae* (mean 4.2 days; range: 1–9), indicated that the life span of ants from mounds was not an impediment to *Apocephalus* spp. parasitism. Therefore, the almost exclusive parasitism of foragers (6.98%) in relation to excavators (0.25%) and also the much higher parasitism (five times) of soldiers collected on the trails compared to parasitism of mound soldiers was unlikely to have been related to host age.

Only two phorid species (*M*. *grandicornis* and *E*. *erthali*) parasitized excavators, whilst all four species studied here attacked the foragers. Furthermore, only *E*. *erthali* was found parasitizing soldier ants. The host preferences of the phorids and the differences in parasitism rates could be due to the relationship between host size and parasitoid size. In fact, there would appear to be a correlation between host size and phorid size, with the larger species of phorids parasitizing larger worker ants. As leaf-cutting ants display morphological polyethism, a division of labor in the colony, when specific tasks are performed by specific sizes of ants [47], phorids preferences could also be correlated to castes. However, as foragers are only slightly larger than excavators, this does not explain why *A*. *attophilus* or *A*. *vicosae* do not parasitize excavators. Therefore, other factors such as oviposition behavior of the different species could explain why these phorids do not parasitize excavators.

*Apocephalus attophilus* exhibits pre-oviposition behavior that involves walking amongst *A*. *laevigata* workers in the foraging area using a highly cautious approach, which apparently helps the phorids avoid detection by the host. When the phorid is very close to the host, it inserts the ovipositor into the ant’s mouthparts, apparently in the basal region of the mandibles, whilst the ant is in the process of cutting the plant material [33]. *Apocephalus vicosae* attacks against *A*. *laevigata* and *A*. *sexdens* occur during the transport of leaf fragments by workers along the foraging trails [22,65]. Female *A*. *vicosae* walk amongst the foraging workers transporting plant fragments and then the phorids jump onto the leaf fragment whilst it is being transported. The fly then walks around the fragment for a few seconds before inserting its ovipositor into the host’s oral cavity [22]. As the excavator caste ants do not cut or carry leaves and probably do not leave the soil mounds, it is unlikely that *A*. *attophilus* and *A*. *vicosae* would be attracted to parasitize them.

Based on knowledge of the attack and oviposition behaviors of *M*. *grandicornis* and *Eibesfeldtphora* spp. against foraging *Atta* spp. workers, and the data available showing the relationship between the size of these parasitoids and their hosts [28,34,52], the parasitism of *M*. *grandicornis* and *E*. *erthali* in *A*. *laevigata* excavators is justifiable. To attack *Atta* spp. foragers, female *Eibesfeldtphora* spp. and *M*. *grandicornis* usually perch on small sticks or leaves in the vicinity of the foraging trails, or near the colony supply holes for a few seconds to a few minutes. They then initiate flight behaviors when they identify suitable individuals to be attacked. It is assumed that at least two of the criteria for the phorids to select the worker to be attacked are the size and mobility of the host.

Low rates of excavator parasitism may be the result of certain difficulties which phorids could face when they attack ants on soil mounds. Firstly, there is a lack of adequate landing sites (such as leaves or plant shoots) for the flies around the craters which form the soil mounds of the *A*. *laevigata* nests [32, 44; MALB personal observation; Fig 1C], necessary for the phorids to observe their hosts prior to attack. Secondly, the soil mounds are agglomerates of loose particles that could make locomotion difficult for the excavators. As mentioned above, the phorid attack sequence is initiated following observation of the host in motion. Furthermore, the excavators spend only part of their time on the surface, and the other part underground, reducing the chances of them being parasitized. Finally, the fact that only two species of phorids (*E*. *erthali* and *M*. *grandicornis*) parasitize excavators, this could be related to the host-parasitoid size relationship as stated previously. In this case, larger (*E*. *erthali*) and smaller (*M*. *grandicornis*) phorids have a preference for larger and smaller leaf-cutting ant excavator workers, respectively.

Interestingly, the size of parasitized foraging workers was greater than the size of non-parasitized excavator workers (Fig 2). This result shows that *A*. *laevigata* foragers transiting on the trails during the period of this study were larger than the excavator ants on the earth mounds. This is probably the reason why among the parasitized workers, the average size of the excavators was smaller than the average size of the foragers. This means that *E*. *erthali* and *M*. *grandicornis* had smaller hosts available during attacks on excavators than when carrying out attacks on foragers. Even so, the parasitism of the excavators by the two parasitoids (*E*. *erthali* and *M*. *grandicornis*) was viable because the limits of the size distribution of these ants on the mounds comprised individuals with adequate sizes to be parasitized by the two flies (Fig 4).

*Eibesfeldtphora erthali* adults that emerged from the host ants were twice the average size of *M*. *grandicornis*. Similarly, the average size of *A*. *laevigata* workers attacked by *E*. *erthali* was almost twice that of workers parasitized by *M*. *grandicornis*. The size of the other species of *Apocephalus* parasitoids in comparison to the size of their hosts were intermediate between *E*. *erthali* and *M*. *grandicornis*. However, although *A*. *attophilus* individuals are smaller than those of *A*. *vicosae*, *A*. *attophilus* hosts were larger on average than *A*. *vicosae* hosts but smaller than those of *E*. *erthali*. Despite being smaller, *A*. *attophilus* had a preference for larger ants. This preference may have evolved to accommodate the development of more than one larva within the host. Furthermore, considering the relationship between host size and parasitoid size, only *E*. *erthali* was identified parasitizing soldiers, correlating the largest of the parasitoid species studied here with the largest ant caste. The lack of parasitism of soldier ants by *A*. *attophilus* and *A*. *vicosae* could be related to the behavior of soldier ants, that do not cut or transport leaves [72; MALB and DJS, personal observations], which as described above, may also reduce the attraction of the excavator caste for the these two phorids.

*M*. *grandicornis*, the smallest phorid species studied here, attacks smaller foragers when compared to those parasitized by *E*. *erthali*. Although soldiers are likely to have a more sclerotized integument than foragers, *E*. *erthali* females are still able to penetrate the integument of this caste, whereas it is unlikely that the structure of the *M*. *grandicornis* ovipositor is capable of performing this task. The dorsal and ventral lobes of the *E*. *erthali* ovipositor are characteristically wide, indicating a greater robustness of this structure [73] when compared to the tubular morphology of the *Myrmosicarius* spp. ovipositor [55]. Although *M*. *grandicornis* has a tubular ovipositor, this fly is half the size of *E*. *erthali* (Fig 3B) and therefore could not oviposit in soldier ants, which are twice the size of excavators (Fig 2), and soldier ant integument is more robust than that of the excavators. Another possible reason for the lack of *M*. *grandicornis* parasitism in soldier ants is the relationship between the size of this parasitoid and the size of the soldiers. According to Elizalde & Folgarait [23], the larval development site in a host ant needs to be large enough for the adequate formation of the phorid larvae, but if too large, then putrefaction could start before the larvae complete their development, which would obviously be a disadvantage.

An important consequence of the coexistence of four species of phorids parasitizing workers of different sizes and different castes is that the parasitoids probably have a combined effect on disrupting normal colony activities and increased the mortality of *A*. *laevigata*. The specificity of these parasitoids for different castes or size classes increases the overall parasitism rates and shows that it is important to maintain the natural biodiversity, as each phorid species will have an influence on colony health.

Two recent studies indicated that *A*. *attophilus* is one of the most promising species for use in biological control programs for leaf-cutting ants [66,74]. However, it is also important to consider the array of phorid species, which taken as a whole, exert a greater effect on leaf-cutting ant populations and disruption of normal foraging behavior. Therefore, it is of interest to consider using more than one species of phorid to control leaf-cutters.

## Supporting information

S1 Data (XLSX)
